# Pleural Effusion Identified by Point of Care Ultrasound (POCUS) in Septic Shock: Impact on Clinical Outcomes

**DOI:** 10.24908/pocus.v9i2.17293

**Published:** 2024-11-15

**Authors:** Erick Joel Rendón-­Ramírez, Andrés Mauricio Morales-­García, Adrián Rendón­-Pérez, Homero Nañez-Terreros, Ricardo Cesar Solis, Alexandra Daniela Magaña­-García, Samantha Medrano­-Juárez, Jose Francisco Caloca-Estrada, Roberto Mercado-­Longoria, Jorge Eduardo Leija­-Herrera, José M Porcel

**Affiliations:** 1 Servicio de Neumología y Cuidados Intensivos,, Hospital Universitario "Dr. José Eleuterio González", Universidad Autónoma de Nuevo León, CIPTR Monterrey, NL MEX; 2 Departamento de Medicina Interna, Hospital Universitario "Dr. José Eleuterio González", Universidad Autónoma de Nuevo León Monterrey, NL MEX; 3 Servicio de Neumología y Cuidados Intensivos, Hospital Universitario "Dr. José Eleuterio González", Universidad Autónoma de Nuevo León, CIPTR Monterrey, NL MEX; 4 Departament of Internal Medicine, Hospital Universitario Arnau de Villanova, IRBLleida Lleida ESP

**Keywords:** Pleural effusion, Septic Shock, Intensive Care, NUTRIC, POCUS

## Abstract

**Aim: **To analyze the association between pleural effusion detected by chest point of care ultrasound (POCUS) and clinical outcomes in patients with septic shock admitted to an intensive care unit (ICU).** Material and methods: **A prospective evaluation of ICU patients with septic shock in whom chest POCUS was performed during the first 24 hours of diagnosis to identify the presence and characteristics of pleural effusion. **Results: **Of 45 patients with septic shock, 17 (38%) had pleural effusion. Mortality (13 vs 17 patients, p=0.44), as well as length of stay in ICU (11.0 vs 6.5 days, p=0.161) were similar among groups. However, there was a significant difference in the modified Nutrition Risk in Critically Ill (mNUTRIC) score between the pleural effusion (5.82±1.13) and non-pleural effusion groups (4.00±2.39, p=0.001). In addition, patients with pleural effusion required more days on mechanical ventilation than those without pleural effusion (10 vs 7, p=0.04). A subgroup analysis of chest POCUS characteristics between surviving and non-surviving patients with pleural effusion identified a higher median size of pleural effusion in the non-surviving group (3±2.16 cm vs 1.9±0.6, p=0.01). **Conclusion: **Pleural effusion in patients with septic shock is associated with high mNUTRIC scores and more days on mechanical ventilation. The larger the pleural effusion, the lower the survival rate.

## Introduction

Pleural effusion is a common clinical condition that occurs in both pulmonary and systemic diseases 1. The frequency of pleural effusion in critically ill patients in the intensive care unit (ICU) has been reported as 7.7% to 62% 2, 3. These figures are probably an underestimate because other severe pulmonary conditions may mask pleural effusion 4. Identifying pleural effusion in the ICU setting is important because it is associated with increased mortality and morbidity 1, 2.

The most common causes of pleural effusion in a series of 82 ICU patients were infectious exudates (43%), followed by noninfectious exudates (33%) and transudates (24%) 3. The prevalence of pleural effusion associated with sepsis, a common condition in the ICU, may be as high as 54% 5.

Over the last decade, chest point of care ultrasound (POCUS) has become an increasingly utilized diagnostic tool in the study of the pleural space and lungs. It is easy to use, noninvasive, inexpensive, and does not expose patients or personnel to radiation. Chest POCUS allows pleural effusion to be identified, characterized, and quantified more accurately than chest X-ray and avoids the inconveniences of computed tomography (CT) 6.

Most of the evidence supporting a higher mortality in ICU patients with pleural effusion is retrospective, and studies using only chest radiographs as the standard imaging test could have underestimated the presence of pleural effusion 2. Currently, chest POCUS has become a standard approach for identifying pleural effusion in critically ill patients. However, evidence regarding the impact of pleural effusion on the clinical outcomes of patients with septic shock is scarce. The objective of this study was to assess the relationship between pleural effusion — ­identified through chest POCUS in ICU patients with septic shock — and various clinical outcomes, including mortality rates, critical care scores, the duration of mechanical ventilation, and the ICU length of stay (LOS).

## Methods

We conducted a prospective, longitudinal, and comparative study of consecutive adult patients with septic shock admitted to the ICU of the Hospital Universitario “Dr. José Eleuterio González” (Monterrey, Nuevo León, México) from May 2019 to January 2020. The study was approved by the Internal Institutional Review Board (IBR) to ensure compliance with the ethical guidelines and patient confidentiality (Ref. NM19-00003). 

Subjects for our study were selected if they met the following criteria: they were 18 years old or above and had been diagnosed with septic shock within 24 hours of being mechanically ventilated. All patients who possessed these attributes were included, regardless of severity assessed using scales or vasopressor administration. Patients were excluded if they had a history of heart failure with New York Heart Association (NYHA) classes III-IV, end-stage chronic kidney disease, chronic liver disease (Child-Pugh score >10), active cancer, chest trauma or chest tube on admission, acute myocardial infarction, and/or cardiogenic shock. 

### Data collection

The data collected included age, sex, comorbidities, etiology of sepsis, mortality in the ICU, LOS in the ICU, days on mechanical ventilation, days under vasoactive amines, chest POCUS characteristics, and ICU scores (see below). A telephone call to patients 90 days after their release from ICU admission enabled the recording of mortality.

Sepsis was defined based on the Sepsis-related Organ Failure Assessment (SOFA) score 7. SOFA scores range from 0 to 24. A score ≥2 in the setting of infection strongly supports the diagnosis of sepsis. Patients with sepsis and persistent hypotension who required fluid and vasoactive resuscitation were considered to have septic shock.

### Chest POCUS 

All individuals were evaluated by chest POCUS using the Mindray R Mobile Trolley equipment model UMT-150p and a convex probe under an abdominal software preset 8. Chest POCUS was performed during the first 24 hours of diagnosis by a pulmonologist with seven years of experience, who was blinded to the clinical data or follow-up. Four thoracic areas per hemithorax were evaluated, including two anterior and two lateral (eight areas in total), with the patient in the supine position. The presence of lung sliding with an A-pattern was considered normal. More than three B-lines, the shred sign or other signs of consolidation, dynamic or static air bronchograms, or pleural effusion were considered abnormal findings. Pleural effusion was described in terms of location, echogenic pattern (simple or complex) 9, volume in mL using the Balik´s formula (volume, ml= measured distance between parietal and visceral pleura in mm x 20) 10, distance in cm between the parietal and visceral pleura, and number of intercostal spaces (ICS) occupied by fluid (1 ICS=small pleural effusion, 2-3 ICS=moderate pleural effusion, ≥4 ICS=large pleural effusion). A pleural effusion of less than 2 cm (distance between the visceral and parietal pleura) was deemed insufficient for aspiration.

The management of pleural effusion followed the standard practices employed by critical care physicians, which considered factors such as pleural effusion size, chest POCUS characteristics, and the results of pleural fluid analyses, if conducted. Chest tubes were used when pleural effusion was homogenously echogenic or complex septated with a high suspicion of an infectious etiology; when macroscopic pus was obtained from pleural effusion aspiration; or when the result of pleural fluid biochemistries supported a complicated infection (pH<7.2, positive Gram stain, glucose < 60 mg/dL) 11, 12, 13. Anechoic pleural effusion patterns with bilateral involvement were managed using an expectant approach, considering volume overload as a potential contributing factor. The authors of this study did not participate in the medical management decisions.

### ICU scores

SOFA, Acute Physiology and Chronic Health Evaluation (APACHE) II, and modified Nutrition Risk in Critically Ill (mNUTRIC) scores were calculated to determine patient severity. The SOFA score evaluates respiratory, cardiovascular, coagulation, central nervous, renal, and liver dysfunction in critically ill patients 14. A higher number of points correlates with a higher hospital mortality (e.g., a SOFA score of 7-9 is associated with 15-20% mortality, while scoring 10-12 is related to 40-50% mortality). The APACHE II is a system that classifies disease severity using 12 different items related to age, previous functional state, and physiological parameters 15. APACHE II scores range from 0-71 points; the higher the score, the higher the risk of hospital death. Finally, mNUTRIC discriminates critically ill patients with the need for aggressive nutrition. The mNUTRIC scale evaluates five items: age, APACHE II score, SOFA score, number of comorbidities, and number of days from hospital admission to ICU admission. A score of 5-9 is considered high and is associated with worse clinical outcomes, indicating that patients are most likely to benefit from aggressive nutrition therapy 16.

### Statistical Analysis

For the descriptive analysis, we looked for normality using the Kolmogorov-Smirnov test for continuous data. Central tendency measures were mean and standard deviation in parametric data, and median and interquartile ranges (IQR) for nonparametric distributed data. For continuous bivariate comparative analyses, the t-test or U-Mann Whitney was used, depending on the data distribution. For categorical variables, Chi-squared or Fisher’s exact tests were performed in accordance with the expected counts of values. For the primary outcome, the overall mortality at 90 days was compared between the patients with and without pleural effusion. Secondary outcomes included LOS in the ICU, days on vasoactive amines, and days on mechanical ventilation. Survival analysis was performed to measure ICU mortality. Kaplan-Meier curves and log-rank tests were used for this purpose. Patients with pleural effusion were analyzed for differences in survival according to the effusion size. Statistical significance was set at p value ≤ 0.05. Analysis was performed using R version 4.0

**Table 1:** Baseline characteristics of the study

	**Pleural effusion(N=17)**	**No pleural effusion(N=28)**	**p value**
Age, years	50.1 (±17.8)	43.3 (±17.5)	0.224
Female	9 (52.9%)	12 (42.9%)	0.727
BMI	28.2 (±4.23)	27.3 (±6.24)	0.563
Apache II score	23.5 (±3.92)	22.3 (±8.67)	0.503
SOFA score	10.2 (±1.38)	9.36 (±3.14)	0.236
mNUTRIC score	5.82 (±1.13)	4.00 (±2.39)	0.001
Fluid balance at ICU admission, mL	595 (±1530)	797 (±1100)	0.639
Arterial hypertension	8 (47.1%)	10 (35.7%)	0.66
Diabetes mellitus	6 (35.3%)	10 (35.7%)	0.92
Obesity hypoventilation syndrome	0 (0%)	4 (14.3%)	0.27
CRP at ICU admission, mg/L	19.6 (±6.70)	17.5 (±7.81)	0.345

Data are expressed as number (%) or mean (SD) unless otherwise specified. Abbreviations: BMI, body mass index; CRP, C-reactive protein; ICU, intensive care unit; mNUTRIC, modified Nutrition Risk in Critically Ill; SOFA, Sepsis-related Organ Failure Assessment.

## Results

A total of 45 patients with a mean age of 45.9 (±17.7) years were included, of whom 21 (46.7 %) were female. Medical history of hypertension and diabetes was reported in 18 (40%) and 16 (35.6%) patients, respectively. The mean Apache II score was 22.7 (±7.22), and the mean SOFA score was 9.67 (±2.63) (Table 1). 

**Table 2:** Isolated infectious agents.

**Microorganisms**	**Pleural effusion(N=17)**	**No pleural effusion(N=28)**
Acinetobacter baumanni	4 (23.5%)	2 (7.1%)
Burkholderia multivorans	1 (5.9%)	0 (0%)
Escherichia coli	1 (5.9%)	2 (7.1%)
Haemophilus influenzae	1 (5.9%)	0 (0%)
Pseudomonas aeruginosa	2 (11.8%)	3 (10.7%)
Stenotrophomonas maltophila	1 (5.9%)	0 (0%)
Streptococcus pneumoniae	1 (5.9%)	3 (10.7%)
Tuberculosis	2 (11.8%)	2 (7.1%)
Clostridioides difficile	0 (0%)	1 (3.6%)
Influenza	0 (0%)	2 (7.1%)
Pneumocystis jiroveci	0 (0%)	1 (3.6%)
Staphylococcus aureus	0 (0%)	2 (7.1%)
No isolation	4 (23.5%)	9 (32.1%)

Isolation sites: tracheal aspiration, fecal samples, and blood cultures.

The most common etiology of septic shock was pneumonia (21, 46.7%), followed by abdominal sepsis (11, 24.4%), soft tissue/burn infection (4, 8.9%), postsurgical meningitis (2, 4.5%), and others (7, 14.5%). The most frequently isolated microorganisms from tracheal aspiration, fecal samples, and/or blood cultures were Acinetobacter baumannii (6, 13.4%), followed by Pseudomonas aeruginosa (5, 11.1%) and Streptococcus pneumoniae (4, 8.9%), and no isolation was obtained in 13 (28.9%) patients (Table 2).

Overall, 17 (37.7%) patients had pleural effusion. There were no differences in the demographic characteristics between patients with and without pleural effusion (mean age 50±17.8 vs 43.3±17.5 years, p=0.22; females 9 [52.9%] vs 12 [42.9%], p=0.72) (Table 1). 

**Table 3:** Chest POCUS characteristics.

**Characteristic**	**Patients with Pleural effusion(n=17)**
**Pleural effusion side **	
Bilateral	3 (17.6%)
Right	4 (23.5%)
Left	10 (58.8%)
**Pleural effusion pattern **	
Anechoic	4 (23.5%)
Homogenously echogenic	6 (35.3%)
Complex septated	5 (29.4%)
Complex non septated	2 (11.8%)
**Pleural effusion size, cm**	3.70 (±2.46)
**Pleural effusion volume, mL**	740 (± 246)
**Number of intercostal spaces occupied by the Pleural effusion**	2.0 (1-3)
**Pulmonary pattern **	
Dynamic air bronchogram	6 (35.3%)
A pattern	2 (11.8%)
AB pattern	3 (17.6%)
B pattern	4 (23.5%)
Others *	2 (11.8%)

Data are presented as numbers (frequencies), means (standard deviations), or medians (interquartile ranges), as appropriate. *static air bronchogram (atelectasis)

### Pleural effusion and clinical outcomes

The most common pleural effusion location was left-sided in ten patients (58.8%) and bilateral in three (17.6%). Pleural fluid was homogenously echogenic in six (35.3%) patients and complex septated in five patients (29.4%). The mean (SD) size of pleural effusion was 3.7±2.46 cm (740±246 ml). These and other pleuropulmonary characteristics of the patients presenting with pleural effusion are shown in Table 3. Pleural effusion was only analyzed in two patients.

The APACHE II score for the pleural effusion group was 23.5±3.92, while it was 22.5±8.67 for the non-pleural effusion group (p=0.53). A statistically significant difference was found in the mNUTRIC scores between the pleural effusion and non-pleural effusion groups (5.82±1.13 vs 4.00±2.39, p=0.001) (Table 1).

**Figure 1:**
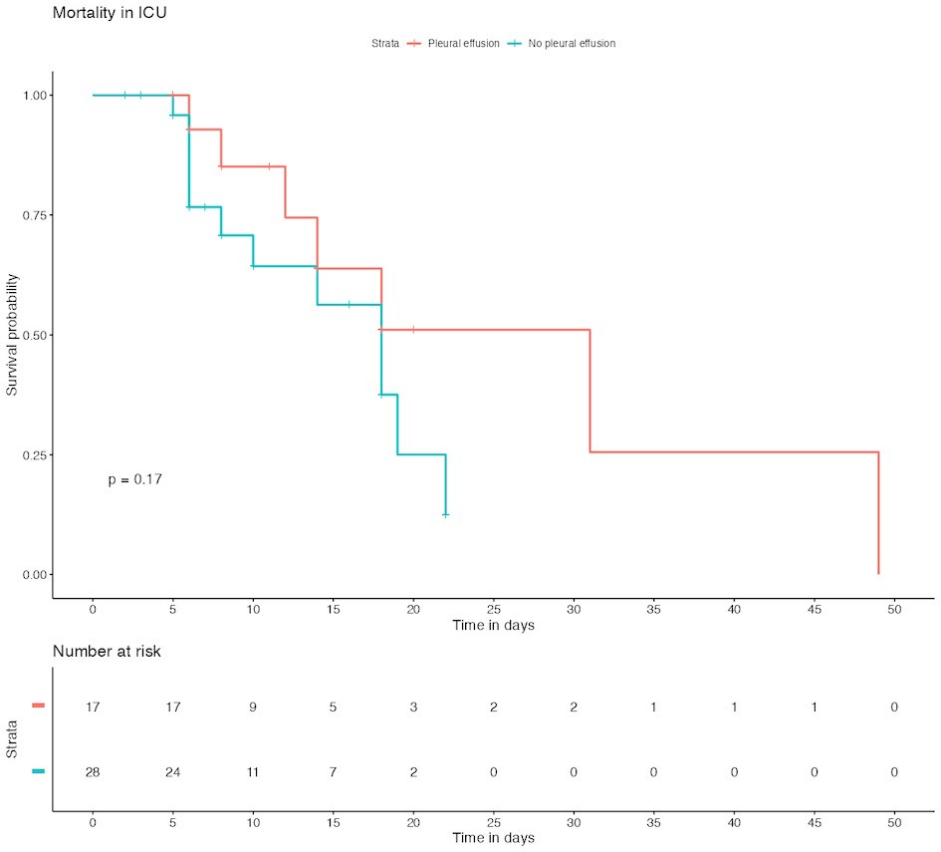
Mortality of septic shock patients with or without pleural effusions.

Overall mortality at 90 days was similar between patients with sepsis presenting with pleural effusion (13, 76.5%) and those without pleural effusion (17, 60.7%, p=0.44). There was no difference in the LOS in the ICU between patients with pleural effusion (11.0 [6.00-8.00] days) and those without pleural effusion (6.50 [5.00-14.5] days, p= 0.161). Notably, pleural effusion was associated with more days on mechanical ventilation (10 8, 9, 10, 11, 12, 13, 14) in the pleural effusion group vs 7 [4.27-12.5] in patients without pleural effusion, p=0.04) (Table 4). The cumulative mortality in the ICU was not significantly different between patients with and without pleural effusion (Figure 1).

A subgroup analysis of the pleuropulmonary characteristics between surviving and non-surviving patients with pleural effusion identified a significantly higher median size of pleural effusion among the latter (3±2.16 cm vs 1.9±0.6, p=0.01). The results of the SOFA score (10.2±1.30 vs 10.0 ± 1.83, p=0.82) and APACHE II score (24.7±2.98 vs 19.8±4.65, p=0.11) were similar in the non-surviving group as compared to the surviving group.

**Table 4:** Clinical Outcomes of the Study Population.

	**Pleural effusion(N=17)**	**No pleural effusion(N=28)**	**p value**
Days with vasopressors	5.00 (3.00-8.00)	4.00 (2.00-5.25)	0.079
LOS at ICU	11.0 (6.00-18.0)	6.50 (5.00-14.5)	0.161
Days on mechanical ventilation	10.0 (8.00-14.0)	7.00 (4.75-12.5)	0.045
Death at 90 days	13 (76.5%)	17 (60.7%)	0.447
Death in the ICU	10 (58.8%)	16 (57.1%)	0.920

Data are presented as numbers (frequencies) or medians (interquartile ranges), as appropriate.

Abbreviations: ICU, intensive care unit; LOS, length of stay.

## Discussion

Pleural effusion has been associated with increased mortality in different pathologies including malignancies, infectious diseases, cardiac diseases, and liver diseases. It is considered a marker of disease severity and advanced organ dysfunction 2, 17. In this study, we found that ICU patients with septic shock and associated pleural effusion had significantly higher mNUTRIC scores and spent more days under mechanical ventilation than those without pleural effusion. Although there was no difference in mortality between the pleural effusion and non-pleural effusion groups, a tendency towards increased mortality in patients with pleural effusion (76.5% vs 60.7%) was noted. This finding contrasts with some studies that have reported an increased mortality in critical care patients with pleural effusion, although they did not analyze a specific group of patients with septic shock 1, 2, 17. It should be highlighted that most of the studies reporting differences in mortality used chest radiography to identify pleural effusion, in contrast with the more sensitive chest POCUS, as we did 1, 3, 17, 18. This enabled us to identify small-to-moderate sized pleural effusion that could have gone unnoticed on chest radiographs, thus potentially affecting mortality outcomes. Chest POCUS has been reported to allow the identification of pleural fluid volumes as small as 5-20 mL, with a sensitivity of 100% when the volume is greater than 100 mL 19, 20, 21. Therefore, it is more precise than chest radiography and has a similar level of accuracy to chest CT, which is the gold standard for the diagnosis of pleural effusion 19, 20, 22.

Moreover, in the sub analysis of patients with pleural effusion, a higher mortality was identified in those with a larger effusion size. This is consistent with studies on other diseases causing pleural effusion that used CT instead of chest POCUS^1^. However, the use of CT implies transportation of the patient to the radiology department, which may be impractical for ICU patients.

Notably, septic shock patients with pleural effusion spent more days on mechanical ventilation. This is in accordance with previous prospective studies, in which patients with pleural effusion also required longer days of intubation [2,23-25]^2^. Mattison et al. studied ICU patients to determine the frequency and importance of pleural effusion using chest radiographs as a reference imaging modality [23]. In contrast to our study, where there were no differences in LOS, it was found that patients with pleural effusion spent more days in the ICU than those without (9.8 vs 4.6). However, in that study, there were important differences in population characteristics between patients with and without pleural effusion, including age, APACHE II scores, and albumin levels [25]. These differences were not observed in our study population, which may have influenced the final LOS.

Most of our patients had small pleural effusion that were not amenable to tapping. No differences were found in SOFA or APACHE scores according to the presence or absence of pleural effusion; however, differences were found in the mNUTRIC score, which was significantly higher in patients with pleural effusion. This indicates that ICU patients with pleural effusion may benefit from aggressive nutritional therapy.

Our study has strengths, particularly the prospective design and systematic assessment of pleural effusion by chest POCUS in patients with sepsis. Moreover, the pulmonologist performing chest POCUS was blinded to the patients’ clinical information. Even though this clinician was an expert in chest POCUS, the acquisition of basic skills for the assessment of pleural fluid is simple, and most pulmonologists currently have adequate training in chest POCUS. The study has certain limitations, including the small sample size, the single center study design, and the absence of systematic pleural effusion sampling. Although the critical care staff was aware of the presence of pleural effusions as they routinely performed chest POCUS, they did not typically perform sampling, with only two instances of thoracentesis. This could be attributed to the fact that many of the pathological ultrasound patterns, such as those that are echogenic or complex septated, occurred in effusions smaller than 2 cm in patients on mechanical ventilation, which posed a high risk of procedural complications. Additionally, in cases of anechoic pleural effusion, the critical care staff deemed it indicative of overload and opted not to perform tapping. Finally, we acknowledge the limitation of the generalizability of the secondary outcomes since no calculation of the proper sample size for this purpose was performed.

## Conclusion

Pleural Effusion identified by chest POCUS in patients with septic shock was associated with high mNUTRIC scores and more days on mechanical ventilation, but mortality was similar to that in patients without pleural effusion. Surviving patients presented with smaller pleural effusion than those who did not. Further studies are required to confirm these results.

## Conflict of interest

Dr. Adrian Rendon reports personal fees and nonfinancial support from GSK, AstraZeneca, Chiesi, and Sanofi. 
